# Speech-specific categorical perception deficit in autism: An Event-Related Potential study of lexical tone processing in Mandarin-speaking children

**DOI:** 10.1038/srep43254

**Published:** 2017-02-22

**Authors:** Xiaoyue Wang, Suiping Wang, Yuebo Fan, Dan Huang, Yang Zhang

**Affiliations:** 1 School of Psychology, South China Normal University, Guangzhou, 510631, China; 2 Center for Studies of Psychological Application, South China Normal University, 510631, China; 3 Guangdong Provincial Key Laboratory of Mental Health and Cognitive Science, South China Normal University, Guangzhou, 510631, China; 4 Guangzhou Rehabilitation and Research Center for Children with Autism, Guangzhou Cana School, Guangzhou, 510540, China; 5 Department of Speech-Language-Hearing Science, University of Minnesota, Minneapolis, MN, 55455, USA; 6 Center for Neurobehavioral Development, University of Minnesota, Minneapolis, MN, 55455, USA

## Abstract

Recent studies reveal that tonal language speakers with autism have enhanced neural sensitivity to pitch changes in nonspeech stimuli but not to lexical tone contrasts in their native language. The present ERP study investigated whether the distinct pitch processing pattern for speech and nonspeech stimuli in autism was due to a speech-specific deficit in categorical perception of lexical tones. A passive oddball paradigm was adopted to examine two groups (16 in the autism group and 15 in the control group) of Chinese children’s Mismatch Responses (MMRs) to equivalent pitch deviations representing within-category and between-category differences in speech and nonspeech contexts. To further examine group-level differences in the MMRs to categorical perception of speech/nonspeech stimuli or lack thereof, neural oscillatory activities at the single trial level were further calculated with the inter-trial phase coherence (ITPC) measure for the theta and beta frequency bands. The MMR and ITPC data from the children with autism showed evidence for lack of categorical perception in the lexical tone condition. In view of the important role of lexical tones in acquiring a tonal language, the results point to the necessity of early intervention for the individuals with autism who show such a speech-specific categorical perception deficit.

Autism spectrum disorder (ASD) is a neurodevelopmental disorder that affects communication and social interaction^1^. Many individuals with ASD show abnormal linguistic profiles in terms of speech perception and production^2^, which may be partially due to a central deficiency in processing complex sounds, especially speech^3^,^4^,^5^,^6^,^7^. One area of research that has intrigued experimental and theoretical investigators studying atypical auditory processing in ASD concerns pitch perception in relation to music and language.

Enhanced pitch percption has been reported in adults and children with autism in contrast to age-matched adults and typically developing (TD) controls in English and Finnish speaking populations^8^,^9^,^10^. A recent comparative analysis revealed markedly different developmental trajectories of pitch discrimination in people with and without ASD^11^. While the TD individuals showed increasing sensitivity to pitch differences with age that was correlated with receptive vocabulary growth, the ASD group had enhanced pitch discrimination in childhood which remained stable in development with no correlation with receptive vocabulary scores. In behavioral tasks, both children and adults with autism outperformed controls in discriminating pure tones as well as pitch patterns in both meaningful spoken sentences andmeaningless vocal sounds^8^,^12^. Studies using the neurophysiological measure known as the mismatch negativity (MMN) response found a complementary pattern of results. Children with autism showed shortened MMN latencies with enlarged amplitudes for pitch contrasts in pure tones^9^,^13^. The MMN reflects pre-attentive automatic detection of acoustic stimulus change independent of attention, which is correlated with perceptual auditory discrimination^14^. Several studies have shown that MMN amplitude is correlated strongly with behavioural parameters such as reaction time and discrimination threshold^15^. Consistent with these results, functional magnetic resonance imaging (fMRI) studies report greater activity in the supramarginal gyrus related to pitch processing in the ASD group in comparison with typically developing controls^16^,^17^,^18^, indicating enhanced pitch perception in ASD at the group level.

Unlike English or Finnish, lexical tones in tonal languages such as Chinese and Thai are defined as a suprasegmental pitch contrast feature that serves a phonemic role and is central to the comprehension and production of speech. Although Chinese infants are able to discriminate lexical tones at 8–10 months of age or even earlier^19^,^20^, this ability does not appear to be fully developed at the age of three^21^. As more than 70% of the world’s languages are tonal languages^22^, it is important to ask the question: Does enhanced pitch perception in ASD have a positive effect on learning a tonal language? While studies on norml adult musicians unanimously indicate a positive transfer effect of their extraordinary pitch processing skills to lexical tone learning^23^, two recent studies on Chinese children with autism have provided both positive evidence for the existence of enhanced pitch perception for the nonspeech stimuli and negative evidence regarding the transfer effect from nonspeech to speech. In a behavioral study^24^, Mandarin-speaking children with autism showed better identification and similar discrimination of melodic contours in comparison with TD controls. However, both identification and discrimination scores were significantly worse in the ASD group when the task switched to speech intonation judgment. A similar pattern was found in an ERP study including both ASD and age-matched TD control groups^25^. In this ERP study, Mandarin-speaking children with autism showed enhanced MMN response for detecting a pitch change in pure tones and nonspeech complex stimuli but reduced MMN to lexical tone contrasts in both spoken words and nonwords. Thus in tonal language speakers with autism, both studies demonstrate domain specificity of enhanced pitch perception for nonspeech stimuli combined with a negative impact on pitch processing in speech.

In our previous study, Yu, *et al*.^25^ discussed the possibility that impaired pitch perception for lexical tones in Chinese children with autism might be due to an underlying phonological processing deficit resulting from enhanced pitch processing for within-category variations in each of the four lexical tone categories in Mandarin Chinese. Due to differences in speech between the numerous individual speakers in their immediate environment, Mandarin-speaking children experience a wide variability in pitch information associated with lexical tones within each lexical tone category. In order to establish stable mental representations of the phonological categories for the lexical tones, the listener needs to develop enhanced sensitivity to between-category contrasts and ignore subtle within-category variations, which is known as categorical perception (CP) for speech sounds^26^,^27^. In categorical perception of lexical tones, the continuously variable pitch information is perceptually mapped onto discrete tonal categories, which has been demonstrated in normal Chinese adults (including musicians) as well as TD children^23^,^27^,^28^,^29^. If the Yu, *et al*.^21^ hypothesis is correct, we would expect to see a CP deficit for lexical tones in Chinese children with autism. In other words, Chinese children with autism would not demonstrate enhanced lexical tone discrimination for between-category stimulus pairs relative to within-category stimulus pairs when pitch differences are physically equated. This hypothesis is in line with previous autism studies showing that individuals with autism show impairment in discriminating higher-level categorical information such as ellipses and faces^30^,^31^,^32^, but intact discrimination of pure tones and colors^8^,^33^. However, our previous report stopped short of providing direct empirical evidence to support this theory^25^.

The present ERP study was specifically designed to address the potential speech-specific CP deficit in Chinese children with autism. In particular, we hypothesized that Mandarin-speaking children with autism and age- and IQ-matched TD controls would show different Mismatch Response (MMR) patterns for within-category and between-category differences across a lexical tone continuum. To further test domain specificity, we used a harmonic nonspeech version of the continuum as a refined acoustic control^27^. The harmonic nonspeech stimuli preserved the same pitch contours of the lexical tone stimuli. A passive listening oddball paradigm was adopted in our study as it has been widely used in developmental research on auditory and linguistic processing in autism^4^,^25^,^34^,^35^,^36^. Because the experimental protocol does not require focused attention or any overt response, the neurophysiological approach can serve as an objective tool to measure auditory discrimination and speech processing^37^,^38^. We were specifically interested in comparing the MMRs to within- and between- category pitch differences in both speech and nonspeech contexts in the two subject groups.

In addition to the conventional ERP latency and amplitude measures, we applied time-frequency analysis to examine trial-by-trial consistency of neural oscillations in selected frequency bands of interest that could drive the MMR activity for speech and nonspeech discrimination. The cortical oscillations are thought to reflect the net excitatory and inhibitory neuronal activities that mediate sensory and cognitive events^39^,^40^,^41^,^42^,^43^,^44^,^45^. Time-frequency analysis on a trial-by-trial basis allows a more detailed examination of what oscillatory activities contribute to or do not contribute to the observed ERP responses that are averaged across trials. Unlike the ERPs, the frequency-specific oscillatory activities are not necessarily time- or phase-locked to an event^46^. Thus trial-by-trial time-frequency analysis may reveal additional information about how various EEG frequencies may reflect multiple neural processes co-occurring and interacting in the service of integrative and dynamically adaptive information processing^47^. The phase-synchronized oscillations survive cross-trial averaging and are evident in the averaged ERPs^42^,^43^. Certain frequency bands have been linked to different neurocognitive process^48^,^49^. In particular, studies have revealed the contribution of the theta frequency band (4–8 Hz) to the neuronal generation of the MMN in frontal and temporal areas^50^,^51^,^52^,^53^,^54^. The theta activity has also been found to be associated with several other cognitive functions including memory encoding, retrieval, and maintenance^55^,^56^. Activity in the beta frequency band (15–30) is thought to play a prominent role in perceptual binding and network interactions across modalities^49^,^57^,^58^. In language studies, beta activity was found to be associated with auditory/lexical memory^59^ as well as vowel representation in an MMN paradigm^60^, and the theta frequency band was found to be important for processing phonemic contrasts^61^. Little is known about the effects of phonemic contrast on theta/beta oscillations during categorical perception of lexical tones and whether there would be between-subject-group differences in the theta and beta activities for the speech and nonspeech conditions.

For the present study, we hypothesized that there would be speech-specific abnormalities in the MMR component and theta/beta frequency bands for processing the within- vs. across-category distinctions in the ASD group, which may reflect problems in speech categorization at the pre-attentive level. As previous neurophysiological studies have also shown atypical attention to pure tones and speech sounds in children with autism^62^,^63^,^64^, the P3a component indexing involuntary attention switching in novelty detection^65^ was also examined to assess whether and how the P3a responses for the speech and nonspeech conditions differed in the two groups of children. With regard to the time-frequency analysis, we hypothesized that the different MMN patterns for within- and across- category lexical tone discrimination in the Mandarin-speaking children with and without autism would be associated with distinct beta/theta phase-locking patterns across the individual trials. In particular, we predicted that Chinese children with autism would not demonstrate enhanced beta/theta activities for the across-category stimulus pairs relative to within-category stimulus pairs when pitch differences are physically equated.

## Results

### Amplitude and latency data for MMR

**Figure 1:**
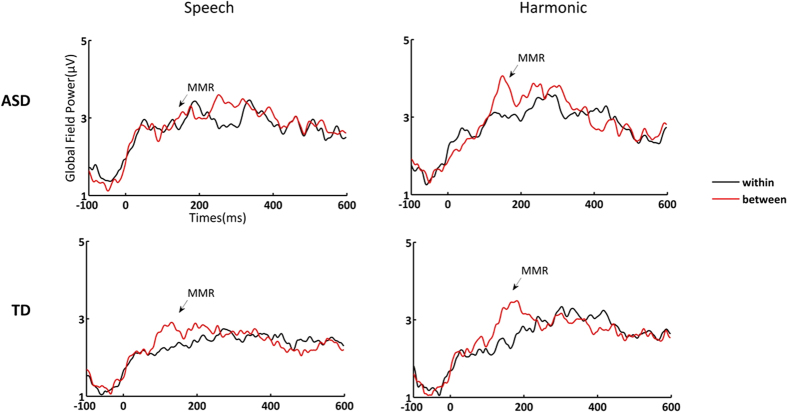
Deviant-minus-standard difference waves for the speech and harmonic conditions.

**Figure 2:**
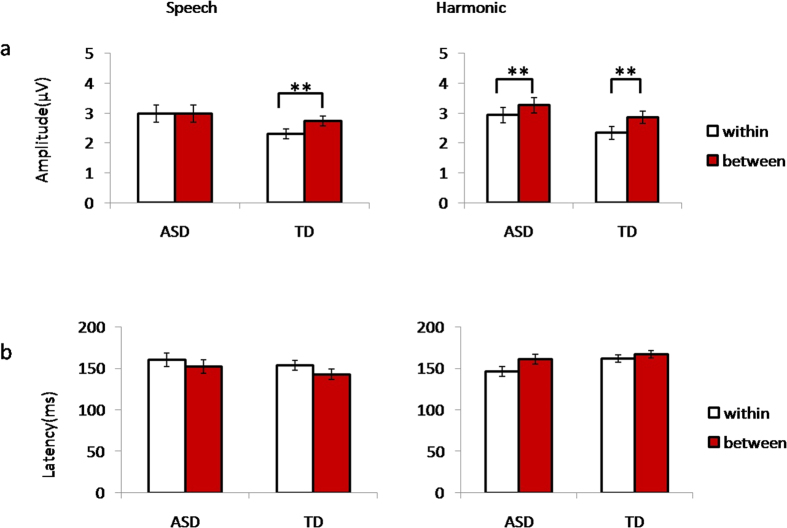
MMR average amplitude (**a**) and latency (**b**) values (vertical bars represent standard error, **P < 0.01). For amplitude values there was an interaction between group and deviant type in the speech condition, and the main effect of deviant type was significant in the harmonic condition. No interactions or main effects were significant for latency values.

For the MMR amplitudes in the speech condition (Table 1 and Figs 1 and 2), there was a significant interaction between deviant type and subject group (*F*(1, 29) = 4.244, *p* = 0.048, partial η^2^ = 0.128), suggesting that neural discriminatory sensitivity to the within- and between- category lexical tone differences depended on the subject group factor. Further two-tailed tests of simple main effects showed that the amplitude of the response to the between-category deviant was greater than that to the within-category deviant in the control group (*t*(14) = −3.379, *p* = 0.004), but not in the ASD group (*t*(15) = 0.302, *p* = 0.767). Moreover, no group effect was found in either the within-category contrast (*F*(1, 29) = 1.911, *p* = 0.190, partial η^2^ = 0.128) or the between-category contrast (*F*(1, 29) = 0.023, *p* = 0.882, partial η^2^ = 0.002). The MMR latencies for the speech condition showed no interaction between group and deviant type (*F*(1, 29) = 0.020, *p* = 0.889, partial η^2^ = 0.000). There was also no main effect of group (*F*(1, 29) = 1.488, *p* = 0.232, partial η^2^ = 0.049) or deviant type (*F*(1, 29) = 0.542, *p* = 0.468, partial η^2^ = 0.018).

For MMR amplitude in the nonspeech harmonic condition (Table 1 and Figs 1 and 2), there was no interaction between deviant type and subject group (*F*(1, 29) = 0.172, *p* = 0.681, partial η^2^ = 0.006). The main effect of deviant type was significant (*F*(1, 29) = 10.21, *p *= 0.003, partial η^2^ = 0.260), indicating that the response to between-category deviants was greater than response to within-category deviants in both groups. The main effect of group was significant (*F*(1, 29) = 4.977, *p *= 0.034, partial η^2^ = 0.146), indicating that ASD group evoked greater responses than control group. MMR latency in the harmonic condition (Fig. 2) showed no interaction between group and deviant type (*F*(1, 29) = 0.033, *p *= 0.857, partial η^2^ = 0.001), as well as no main effect of group (*F*(1, 29) = 0.589, *p *= 0.448, partial η^2^ = 0.019) or deviant type (*F*(1, 29) = 1.226, *p* = 0.277, partial η^2^ = 0.038).

### Amplitude and latency data for P3a

For the P3a amplitudes in the speech condition, the difference in the P3a component between deviants and standards was significant for both types of deviants in both subject groups (*t*_ASD-within_(15) = 9.185, *p *< 0.001; *t*_ASD-between_(15) = 6.99, *p *< 0.001; *t*_TD-within_(14) = 15.873, *p *< 0.001; *t*_TD-between_(14) = 18.65, *p *< 0.001). However, there was no main effect of group (*F*(1, 29) = 2.295, *p *= 0.141, partial η^2^ = 0.073) or deviant type (*F*(1, 29) = 1.055, *p *= 0.313, partial η^2^ = 0.035), and there was also no interaction between group and deviant type (*F*(1, 29) = 0.642, *p *= 0.430, partial η^2^ = 0.022). The results for the harmonic condition were similar to the speech condition (Fig. 1). While the P3a effects were significant (*t*_ASD-within_(15) = 11.14, *p *< 0.001; *t*_ASD-between_(15) = 6.99, *p *< 0.001; *t*_TD-within_(14) = 13.32, *p *< 0.001; *t*_TD-between_(14) = 18.65, *p *< 0.001). There were no main effect of group (*F*(1, 29) = 0.825, *p *= 0.371, partial η^2^ = 0.028), deviant type (*F*(1, 29) = 0.016, *p *= 0.902, partial η^2^ = 0.001), or interaction between group and deviant type (*F*(1, 29) = 0.491, *p *= 0.489, partial η^2^ = 0.017).

### Inter-trial phase coherence findings

**Figure 3:**
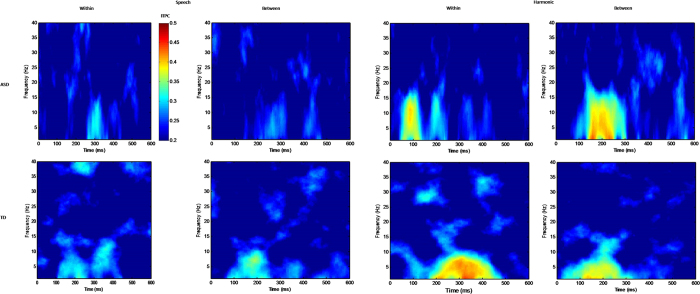
Neural oscillatory response to speech and nonspeech sounds.

Table 2 shows the trial-by-trial phase locking values for the theta and beta bands in response to the within/between-contrast stimuli in both speech condition and harmonic condition. The ITPC plots were shown in Fig. 3.

**Figure 4:**
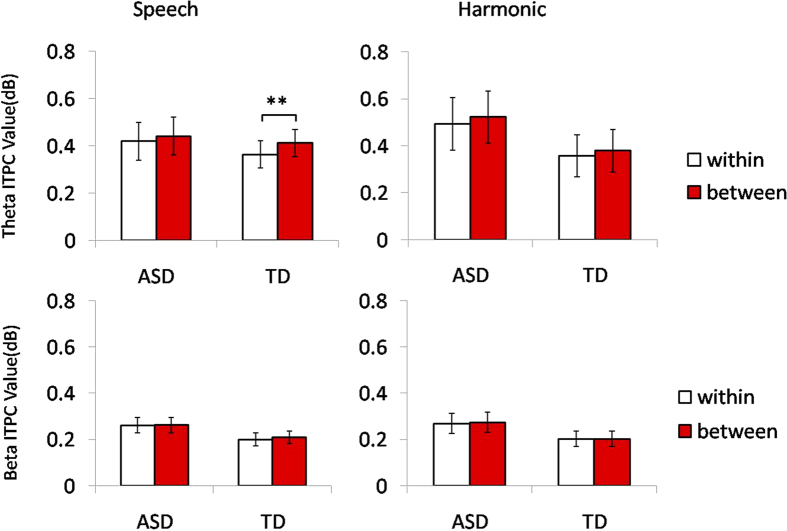
Theta/Beta ITPC values (vertical bars represent standard error, **P < 0.01).

For the theta band in the speech condition (Table 2 and Figs 3 and 4), there was no interaction between deviant type and group (*F*(1, 29) = 1.071, *p* = 0.309, partial η^2^ = 0.036). The main effect of deviant type was significant (*F*(1, 29) = 7.551, *p *= 0.010, partial η^2^ = 0.207), indicating that trial-to-trial phase locking for between-category MMRs was consistently greater than that for within-category deviants. The main effect of group was significant (*F*(1, 29) = 6.166, *p *=* *.019, partial η^2^ = 0.175), indicating that ASD group had higher ITPC values than control group. Further planned t-tests showed that the ITPC values of the theta band for the between-category MMRs was greater than those for the within-category MMRs in the control group (*t*(14) = −3.231, *p* = 0.006), but there was no such effect in the ASD group (*t*(15) = −1.074, *p* = 0.300). The theta band in the harmonic nonspeech condition (Table 2 and Figs 3 and 4) showed no interaction between group and deviant type (*F*(1, 29) = 0.047, *p* = 0.830, partial η^2^ = 0.002). There was also no main effect of deviant type (*F*(1, 29) = 1.917, *p* = 0.177, partial η^2^ = 0.062). Like the speech condition, the main effect of group was significant (*F*(1, 29) = 62.412, *p* < 0.001, partial η^2^ = 0.683), indicating an overall higher trial-by-trial phase-locking of mismatch response in the ASD group than in the control group.

For the beta band in the speech condition (Table 2 and Figs 3 and 4), there was no interaction between deviant type and group (*F*(1, 29) = 0.378, *p* = 0.543, partial η^2^ = 0.023). There was also no main effect of deviant type (*F*(1, 29) = 0.669, *p* = 0.420, partial η^2^ = 0.023). Consistent with the theta activity, the main effect of group was significant (*F*(1, 29) = 38.099, *p *< 0.001, partial η^2^ = 0.568), indicating that ASD group had greater ITPCs than control group. The beta band in the harmonic nonspeech condition (Table 2 and Figs 3 and 4) showed no interaction between group and deviant type (*F*(1, 29) = 0.097, *p *= 0.758, partial η^2^ = 0.003), as well as no main effect of deviant type (*F*(1, 29) = 0.149, *p *= 0.702, partial η^2^ = 0.005). Consistent with the speech condition, we observed significant group differences with greater ITPC values in the ASD group than in the control group (*F*(1, 29) = 42.146, *p *< 0.001, partial η^2^ = 0.592).

## Discussion

### MMRs show speech-specific deficit in categorical perception in autism

The present ERP study employed well-controlled synthesized speech and nonspeech stimuli with equivalent acoustic differences for within- and across- category contrasts to investigate whether Mandarin-speaking Chinese children with autism would show a speech-specific deficit in categorical perception of lexical tones. In the speech condition, the TD control group showed typical enhanced neural sensitivity to the between-category deviant relative to the within-category deviant whereas the ASD group had equivalent MMRs for the two types of deviants. The MMR patterns for the lexical tone in the TD control group were consistent with previous reports on categorical perception of lexical tones in normal adults and children^27^,^28^, indicating that phonological representations of lexical tones in 10-year-old typically developing Chinese children are similar to those in healthy adults^28^. The lack of differentiation in MMRs for within- and between- category contrasts in the autism group confirmed our hypothesized deficit in categorical perception of lexical tones. More importantly, our data revealed that one possible cause for the CP deficit was enhanced within-category MMRs in the ASD group in comparison with the TD control group. This pattern of greater within-category perceptual sensitivity was predicted on the basis of studies finding that discrimination of pure tones and more complex musical stimuli are enhanced in autism^12^,^66^, and further suggests that children with ASD may be more sensitive than controls to the acoustic (as opposed to linguistic) stimulus features at the pre-attentive level. According to the Weak Central Coherence (WCC) theory^67^ and the Enhanced Perceptual Functioning (EPF) theory^68^,^69^, individuals with ASD show a local processing bias in the auditory mode (including pitch sensitivity) combined with an over-developed neural network for low-level perceptual analysis.

The lack of CP for lexical tones in the Chinese children with autism implies that, unlike the TD controls, individuals with autism have not fully established the phonemic boundaries in the acoustic space of fundamental frequency variations and thus have not acquired full phonological knowledge of the different lexical tone categories. Given the fundamental role of lexical tones in a tonal language such as Mandarin Chinese, this phonological processing deficit at the age of ten is alarming in that it could be a substantial impediment to proper language development and efficient speech communication. Assuming that MMRs reflect speech discriminatory sensitivity that is preemptively subordinate to the development of language-specific phonemic representations in the first year of life^4^,^38^, the lack of CP for lexical tones in autism may also be accounted for by top-down mechanisms^70^. For instance, Chinese children with autism may not have developed the prototypical representations of the lexical tone categories to the extent that there is no perceptual magnet effect that would reduce within-category discrimination around the prototypical sound in comparison with a non-prototypical sound^71^.

In order to determine whether the CP deficit reflects abnormality in acoustic or phonological processing, the nonspeech condition using harmonic stimuli was designed. In the nonspeech condition, both subject groups showed similar enhanced neural sensitivity for the between-category contrast. Thus there does not appear to be a CP deficit in the nonspeech domain for Chinese children with autism. In other words, the CP deficit for lexical tones in autism is more likely a phonological processing problem rather than an acoustic processing deficit^25^,^35^,^72^. In cross-language studies of normal listeners, it has been shown that speech perception can be selectively compromised by language learning experience without a parallel effect in auditory processing of nonspeech stimuli that share the essential spectral features of the speech sounds^73^. For example, the normal adult Japanese speakers who had difficulty in identifying and discriminating the third-formant (F3) differences (rising vs. falling) in the English /l-r/contrast did not show any CP deficit when the exact F3 information was extracted from the syllables and tested^74^. But unlike this cross-language example in which the Japanese language does not have the target /l-r/ contrast, the underlying mechanisms for the problem in the acquisition of phonological knowledge can be quite different in autism. We suspect that the early learning experience for lexical tones in the Chinese children with autism may be compromised by their social deficits that could interfere with normalizing and representing the multiple co-existing dimensions of linguistic and paralinguistic (such as speaker age, gender, and affective mood) information in the speech signal, leading to the relatively poor development of the phonological categories for the lexical tones and consequently the CP deficits for the same pitch information in the speech context.

In normal subjects, categorical perception of pure tones and complex tone stimuli (e.g., tones with rising vs. falling fundamental frequency) has been previously reported^8^,^27^. The similar results between the nonspeech and speech conditions for the TD controls could be explained by the shared processing mechanisms for pitch perception –the language learning experience exerts a strong influence on the perception of acoustic features such as pitch perception for lexical tone categorization, and this influence could be extended to processing nonspeech sounds^27^,^75^,^76^. Such shared mechanisms appear to be constrained by domain-specific enhanced pitch processing for non-speech sounds in Chinese children with autism.

Despite the significant MMR amplitude differences between the two groups of subjects in the speech condition, our MMR latencies for the speech condition showed no main effect of group as well as no interaction between group and deviant type. These results are consistent with previous studies on children with autism^12^,^37^ and cross-language studies on categorical perception of lexical tones in normal adult listeners^27^,^28^,^29^. As the lexical tones unfold over the entire syllable, it is possible that the MMR latency may be quite variable across individual listeners and thus become less sensitive to the between-group differences than expected for ASD vs. TD comparison or native vs. nonnative comparison.

Taking the MMR results from the two conditions together, we have found ERP evidence that Chinese children with autism have impaired pitch perception for lexical tones at the group level, which appears to be speech-specific and can be attributed to an underlying phonological processing deficit with impaired phonological representations of lexical tones accompanied by enhanced pitch processing for within-category variations in an acoustic mode.

### P3a component shows no different patterns for small acoustic deviations in autism

For both types of stimuli, there was no significant difference in the P3a response between the two groups of subjects. Our results are similar to Xi, *et al*.^27^ but differ from Yu, *et al*.^25^. The P3a typically reflects attentional orientation or shift towards the stimulus change, which becomes prominent when the difference is large^77^. In the Yu, *et al*.^25^ study, the lexical tone contrasts were based on naturally produced words or nonsense syllables, which could be compared to the endpoints of our 10-inteval continuum. In our study, similar to Čeponienė, *et al*.^62^ and Xi, *et al*.^27^, the physical difference between the standard and deviants was much smaller. Moreover, the standard sound for our double oddball paradigm was Stimulus #5, which was a nonprototypical speech sound close to the phonetic boundary region in our continuum. We suspect that these experimental factors may have minimized differences in arousal and attentional orienting resulting from acoustic differences or other factors such as semantic or social significance. As a result, we did not observe significant P3a differences between the two subject groups in any of the comparisons.

### Theta band activity reflects the presence or absence of categorical perception of lexical tones in the two subject groups

The time-frequency analysis revealed a new aspect of cortical activities responsible for the speech-specific CP deficit in autism. Our results show that theta band activity modulates categorical perception of lexical tones in control group rather than in ASD group. Moreover, the time-frequency analysis results revealed cortical network activation patterns that varied across conditions. Consistent with the MMR results, there was a significant ITPC difference in theta activity for the MMRs of within- vs. across-category contrasts in the control group. Our data further showed equivalent ITPC values in theta band for the two types of deviant stimulus in speech stimuli in ASD group. For harmonic speech stimuli, both ASD group and control group showed similar phase locking across trials in theta band for the across-and within-category contrasts. Although both conditions involved the acoustic presentation of Tone 2 or Tone 4, sensitivity of CP in theta activity (with enhanced across-category discrimination relative to within-category discrimination) was only observed in the speech condition for the TD group. We did not find significant beta activity differences between the two subject groups in any of the comparisons except for the overall group effect with the ASD group showing higher ITPC values than the TD group regardless of stimulus condition, which might contribute to the enhanced sensitivity to pitch information in ASD as previously reported in the literature. Thus, enhanced across-category discrimination for lexical tones in the TD group appears to be associated with stronger phasing locking across trials in the theta activity (4–8 Hz), which corresponds to oscillatory periods in the range of 125–250 ms in line with the duration of our stimuli. By contrast, phase locking across trials in the theta band does not appear to contribute to the within- and across- category distinction in the ASD group. We suspect that learning experience as well as pathological conditions could fundamentally alter the underlying neural network with dedicated and frequency-specific oscillatory activities for the efficient processing of the critical acoustic and phonological cues in service of the native language.

Our results showing the lack of CP for lexical tones in theta activity in the Chinese children with autism are consistent with previous findings that reveal the importance of the theta frequency band in the pre-attentive neural processing of auditory deviant stimuli^42^,^51^,^52^,^53^,^54^,^78^. Our results differ from Bidelman^79^ and Scharinger, *et al*.^60^, in which the beta band activity was correlated with categorical perception of vowel sounds. In the Bidelman^79^ study, an identification task were used for the /a/-/u/ vowel continuum. In Scharinger, *et al*.^60^ study,sounds from /æ/-/I/ and /ε/-/I/ continuua were used in an oddball paradigm. Thus both of these studies involved detection of steady formant differences at the segmental level in normal adult listeners. However, the speech stimuli in our study involved time-varying frequency glides (rising vs. falling) beyond the segmental level. We suspect that the neural oscillation patterns may be affected by phonetic features and acoustic features such as duration of the critical acoustic cue for eliciting the mismatch response.

Taking the ERP waveform results and neural oscillation results from the two conditions together, we found that spectral information in speech and nonspeech stimuli may be processed independently rather than executed as a shared process depending on subject characteristics and stimulus properties. The overall patterns of our findings are consistent with other studies on categorical perception in autism. For example, CP of complex visual stimuli such as ellipses and faces was found to be impaired in autism^30^,^31^,^32^ while the discrimination of pure tones and color was normal^8^,^33^.

### Limitations and Future Directions

The present CP study incorporated two important improvements in experimental design from our previous study^25^. First, our CP study employed subject groups that were age-matched and IQ-matched. Second, our CP study used carefully-controlled synthetic stimuli for the within- vs. across- category contrasts in both the speech and nonspeech conditions. While we found the first evidence for impaired CP for lexical tones in Chinese children with autism, our study is not without its limitations. One outstanding limitation is the lack of behavioral data to verify the CP deficit at the individual subject level. This lack of behavioral data appears to be quite common in MMN studies on children with autism due to practical challenges in implementing behavioral protocols for synthetic speech stimuli^25^. However, it is important to acknowledge that a pre-attentive mismatch response and a behavioral judgment that requires focused attention and an overt response do not necessarily reflect the same sensory and cognitive processes. More importantly, as the MMN measure has been highlighted as a potential clinical biomarker^80^, it is necessary to conduct further studies to test the robustness of the relationship between brain and behavioral measures at the individual subject level.

Our results confirmed the hypothesis from the previous Yu, *et al*.^25^ study that lexical tone processing impairment in Chinese children with autism has its root cause as a phonological deficit in categorical perception. Given the fundamental role of lexical tones for learning and using a tonal language, it is necessary to consider the development of feasible training methods for early intervention. Before implementing such an intervention, it will be important to identify the age at which a clear CP deficit for lexical tone emerges in tonal language speaking children with autism and have a better understanding of how pervasive this problem is. In this regard, future research could benefit from a longitudinal design with a much larger subject sample to identify developmental changes. Although musical experience has been shown to have a positive effect on the learning of lexical tones^23^,^81^, it remains unknown how to best implement musical training independently of or combined with speech training to improve categorical perception by suppression of within-category discriminatory sensitivity and enhancement of between-category sensitivity.

Although the current study is confined to lexical tone processing deficits in Chinese children with autism, our findings add to the recent literature^24^,^25^ that points to the existence of domain-specific pitch perception deficit in autism in relation to the tonal language background. From a theoretical perspective, an important issue regarding atypical auditory processing associated with ASD is to determine whether it reflects domain-general basic auditory sensitivity of enhanced spectral resolution or whether this auditory sensitivity is shaped by language experience such that language-specific patterns of atypicalities would emerge in children with ASD who speak different languages. Given that different languages employ different acoustic cues for phonological representations (for instance, vowel length is used as a phonemic contrast in Finnish and Japanese but not in Chinese or English), further studies could examine other aspects of auditory hyper- or hypo- sensitivity such as temporal processing impairment^37^ in ASD to determine whether similar domain-specific processing issues in autism exist in relation to effects of language learning experience/deficit for the proper acquisition of phonological categories.

## Method

### Participants

The reported study was conducted with approval from the institutional review board of South China Normal University, and in compliance with the 1964 Helsinki declaration and its later amendments ethical standards. All children’s parents gave full, informed, written consent.

Two groups of children participated in this study: 16 children with autism (ASD group, average age 10.4), and 15 typically developing controls (TD group, average age 10.3) matched on age and IQ scores. Children in the ASD group were recruited from Guangzhou Cana School (Guangzhou Rehabilitation Research Center for Children with Autism). The clinical diagnosis of autism was established according to the DSM-V criteria for autistic disorder^2^. We confirmed diagnoses using the Chinese version of the Gilliam Autism Rating Scale–Second Edition^82^. We chose to use the GARS-2 because Chinese versions of other standardized diagnostic instruments, i.e., the Autism Diagnostic Interview –Revised^83^ and the Autism Diagnostic Observation Schedule^1^, have not been officially validated or widely adopted in China^84^,^85^. The GARS-2 has previously been used for this purpose in published autism studies conducted in China^86^,^87^. Stereotyped Behaviors, Communication, and Social Interaction, three subtests of GARS-2, are based on the DSM-IV-TR and Autism Society of America (1994) criteria for autism. Children in the TD group were recruited from all classes of a regular local primary school after screening for language background, medical history of speech-language-hearing problems and psychiatric/neurological disorders. All of the children had normal hearing (<20 dB HL) in standard audiometric screening with pure tones (250~6000 Hz). All of them were right-handed. Non-verbal IQ scores (Raven’s Standard Progressive Matrices) were obtained to verify that there were no significant differences between the two groups. See Table 3 for a summary of the participant characteristics.

### Stimuli

**Figure 5:**
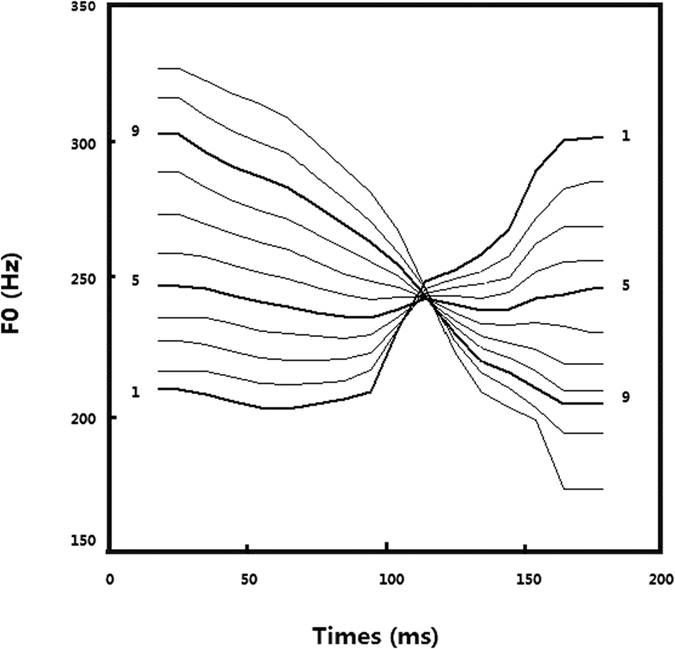
The F0 contour continuum for the speech and nonspeech stimuli. Fundamental frequency patterns of the tonal continuum from the high rising Tone 2 to the falling Tone 4. Stimuli 1, 5 and 9 used in the ERP experiment are marked.

Three speech stimuli were chosen from a 10-step lexical tone continuum used in previous CP studies^27^,^28^ in order to have both a between-category (or across-category) stimulus pair (5 and 9) and a within-category stimulus pair (1 and 5) equated for acoustic distance (Fig. 5). The continuum represented two natural Mandarin Chinese monosyllables at the two end points, /ba2/ and /ba4/, which differed in pitch contour (Tone 2 is the high rising tone, and Tone 4 is the falling tone). As described in the previous publications, the raw stimuli were recorded from a female native Mandarin Chinese speaker. They were digitally edited to have the duration of 200ms. Pitch tier transfer was performed using the Praat software (http://www.fon.hum.uva.nl/praat/) to isolate the lexical tones and keep the rest of the acoustic features identical. A morphing technique was applied in Matlab (Mathworks Corporation, USA) using STRAIGHT software (Kawahara, Masuda-Katsues, & de Cheveigne, 1999) to create the 10-interval speech continuum. The between- and within- category contrasts had equal steps of acoustic change in fundamental frequency along the continuum.

To test whether the hypothesized effects were speech-specific, nonspeech stimuli were generated to match the speech stimuli in terms of fundamental frequency (pitch), amplitude and duration parameters. Spectral components differed between the speech and nonspeech stimuli. In particular, harmonics (1, 3, 6, 7, 8, and 12) of the fundamental frequency were kept, and other harmonics were removed to create the nonspeech percept. All the stimuli were normalized to have equal average root-mean-square (RMS) intensity. See Xi, *et al*.^27^ for more details of synthesis for the speech and nonspeech stimuli; also see Zhang, *et al*.^28^ for a study on Chinese children with dyslexia using similar stimulus materials. Behavioral identification results from normal native Mandarin Chinese speaking subjects from the same previous study were used to select the three stimuli to represent the between- and within- category contrasts. Both Stimuli 1 and 5 on the lexical tone continuum were perceived by the native speakers as Tone 2 and thus was chosen for the within-category contrast. Stimulus 9 was perceived as Tone 4^27^, and Stimuli 5 and 9 were chosen to represent the across-category contrast.

### ERP Procedure

The stimuli were presented binaurally with earphones in a double oddball paradigm following the procedure of previous CP studies^27^. In particular, the standard stimulus was #5 in the continuum; #9 (between-category) and #1 (within-category) were used as the two deviants. The within-category deviant and between-category deviant were presented pseudo-randomly among standards; each had a probability of occurrence of 10%, and adjacent deviants were separated by a minimum of 4 standards. A total of 600 stimuli were presented, each with a duration of 200 ms and a stimulus-onset-asynchrony (SOA) of 1000 ms. The sound intensity level was at approximately 75 dB SPL. Participants were instructed to ignore the presented sounds while watching a muted movie, which was shown with subtitles.

### Data recording and analysis

Continuous EEG was recorded using BrainAmps DC consisting of 32 channels at 500 Hz sampling. The BrainAmps DC system included electrodes next to and below the eyes for recording horizontal and vertical eye movements. The impedance of each electrode was kept below 10 KΩ. The left mastoid electrode was set as the reference, and the AFz electrode served as ground. For the ERP waveform analysis, the raw data were digitally filtered offline with a 1~30 Hz bandpass filter and segmented for epochs of 700 ms duration including a 100 ms baseline prior to the onset of each stimulus. Recorded trials with eye blinks or other artifactual activities beyond the range of −80 μV~80 μV were rejected. Only the standards immediately preceding a deviant was used for averaging and subtraction to keep the number of standard and deviant trials equivalent. There were at least 40 deviant trials accepted in each condition. The MMRs and P3a were derived from the deviant-minus-standard difference ERP waveforms in each condition. MMR and P3a were defined as the response deflection within 100–200 ms post stimulus and 250–400 ms post stimulus respectively. The Global Field Power (GFP) was calculated as a measure of the magnitude of the MMR^88^. Unlike the waveform peak analysis at selected electrodes, the GFP provides an objective assessment of spatial scalp distribution in terms of the standard deviation of potential values for all electrodes at any sampling point in the epoch window^89^.

To understand how trial-by-trial neural oscillatory activities contributed to the generations of the MMRs, time-frequency analysis was completed using the subtracted MMR waveforms (deviant–standard preceding deviant with a bandpass filter of 1–40 Hz) for each accepted deviant trial at electrode Cz with the *newtimef* function in EEGLAB^90^. A short-term Fourier Transform (STFT) with Hanning window tapering^41^, which is recommended for the analysis of low frequency activities, was adopted for the calculation of inter-trial phase coherence (ITPC) of theta and beta band activities. The ITPC measure for a chosen latency ranges from 0 (indicating absence of synchronization across trials) to 1 (indicating perfect synchronization). The time window used for our time-frequency analysis represented the entire analysis epoch, including the pre-stimulus baseline from −100 to 700 ms, and estimated frequencies were from 1 to 40 Hz with a step interval of 0.5 Hz^41^. Automatic artifact rejection criteria were set at ±80 μV. Based on grand mean ERP waveforms, search windows relative to the pre-stimulus interval were determined for MMR at 100–200 ms for both conditions.

Separate two-way group (TD/ASD) × deviant type (within-category/between-category) repeated measures ANOVAs were conducted for each of the dependent variables (mean amplitude and mean latency) for both the MMR/P3a components and band spectral power related to MMR in both the speech and nonspeech (harmonic) conditions. Further tests of simple effects (in the case of significant interaction) and planned two-tailed t-tests were also conducted to verify whether there were significant within- vs. across- category differences for each subject group in the speech and nonspeech conditions.

## Additional Information

**How to cite this article**: Wang, X. *et al*. Speech-specific categorical perception deficit in autism: An Event-Related Potential study of lexical tone processing in Mandarin-speaking children. *Sci. Rep.* **7**, 43254; doi: 10.1038/srep43254 (2017).

**Publisher's note:** Springer Nature remains neutral with regard to jurisdictional claims in published maps and institutional affiliations.

## Figures and Tables

**Table 1:** MMR Mean Amplitude and Latency Data in Children with Autism and TD Controls.

Condition	Amplitude (μV) (*SD*)		Latency (ms) (*SD*)	
	Autism	TD Control	Autism	TD Control
Speech				
within	2.94 (1.55)	2.28 (0.48)	160 (35)	154 (35)
between	2.86 (1.56)	2.88 (0.82)	152 (37)	143 (30)
Harmonic				
within	2.83 (0.97)	2.35 (0.76)	146 (23)	162 (36)
between	3.41 (1.07)	2.76 (0.90)	161 (25)	167 (31)

**Table 2:** Theta and Beta ITPC Value in Children with Autism and TD Controls.

Condition	Theta (*SD*)		Beta (*SD*)	
	Autism	TD Control	Autism	TD Control
Speech				
within	0.42 (0.06)	0.36 (0.05)	0.26 (0.03)	0.20 (0.02)
between	0.44 (0.07)	0.41 (0.06)	0.26 (0.04)	0.21 (0.03)
Harmonic				
within	0.49 (0.07)	0.36 (0.05)	0.27 (0.04)	0.20 (0.03)
between	0.52 (0.09)	0.38 (0.07)	0.28 (0.04)	0.20 (0.04)

**Table 3:** Descriptive Characteristics of the Sample that were Matched in Age and Nonverbal IQ Scores.

	Autism				TD Control				*F*	*p* value
	*n*	*M*	*SD*	Range	*n*	*M*	*SD*	Range		
Age	16	10.4	1.27	9–13	15	10.3	1.55	8–13	0.273	0.605
Nonverbal IQ		83.7	11.7	75–117		86.3	6.61	75–96	0.369	0.548
